# *Malassezia* Intra-Specific Diversity and Potentially New Species in the Skin Microbiota from Brazilian Healthy Subjects and Seborrheic Dermatitis Patients

**DOI:** 10.1371/journal.pone.0117921

**Published:** 2015-02-19

**Authors:** Renan Cardoso Soares, Marcelo Bergamin Zani, Ana Carolina Belini Bazán Arruda, Lucia Helena Fávaro de Arruda, Luciana Campos Paulino

**Affiliations:** 1 Centro de Ciências Naturais e Humanas (CCNH), Universidade Federal do ABC (UFABC), Santo André, SP, Brazil; 2 Hospital e Maternidade Celso Pierro, Dermatologia, Campinas, SP, Brazil; The University of Hong Kong, HONG KONG

## Abstract

*Malassezia* yeasts are part of the resident cutaneous microbiota, and are also associated with skin diseases such as seborrheic dermatitis (SD). The role these fungi play in skin diseases and why they are pathogenic for only some individuals remain unclear. This study aimed to characterize *Malassezia* microbiota from different body sites in healthy and SD subjects from Brazil. Scalp and forehead samples from healthy, mild SD and severe SD subjects were collected. Non-scalp lesions from severe SD patients were also sampled. 5.8S rDNA/ITS2 amplicons from *Malassezia* sp. were analyzed by RFLP and sequencing. Results indicate that *Malassezia* microbiota did not group according to health condition or body area. Phylogenetic analysis revealed that three groups of sequences did not cluster together with any formally described species, suggesting that they might belong to potential new species. One of them was found in high proportions in scalp samples. A large variety of *Malassezia* subtypes were detected, indicating intra-specific diversity. Higher *M. globosa* proportions were found in non-scalp lesions from severe SD subjects compared with other areas, suggesting closer association of this species with SD lesions from areas other than scalp. Our results show the first panorama of *Malassezia* microbiota in Brazilian subjects using molecular techniques and provide new perspectives for further studies to elucidate the association between *Malassezia* microbiota and skin diseases.

## Introduction

Fungi from *Malassezia* genus are lipophilic yeasts that inhabit skin from warm-blooded animals, including humans [1,2]. Molecular based studies have been showing that *Malassezia* yeasts are the most abundant fungi living on the human skin [3,4]. Although members of the healthy skin microbiota, these fungi are also associated with skin diseases, such as atopic dermatitis, psoriasis, pityriasis versicolor, dandruff and seborrheic dermatitis (SD) [5,6].

SD is a highly prevalent condition that affects mostly sebum rich skin sites, such as scalp, forehead, nose, chest and upper back causing skin desquamation, itching and reddish lesions [2], as well as having social and psychological negative impact [7,8]. The role *Malassezia* yeasts play in the development of SD is poorly understood. It also remains unclear why *Malassezia* organisms are pathogenic for only some individuals as they are also present in healthy skin. Among *Malassezia* species, *M*. *globosa* and *M*. *restricta* are the most commonly found species occurring in large proportions on human skin in both healthy individuals and subjects with skin diseases [4,9–13].

*Malassezia* genus underwent many taxonomic revisions in the last 25 years. Up until 1995, three species were accepted: *M*. *furfur*, *M*. *pachydermatis* and *M*. *sympodialis* [14]. The detection of different variants through rDNA sequencing [15] led to a taxonomic revision that resulted in 4 new species based on morphology, ultrastructure, physiology and also molecular data—*M*. *globosa*, *M*. *restricta*, *M*. *obtusa* and *M*. *slooffiae* [16]. Subsequently, other species were described, isolated from humans—*M*. *dermatis* [17], *M*. *japonica* [18] and *M*. *yamatoensis* [19]; and other animals—*M*. *nana* [20], *M*. *caprae* [21], *M*. *equina* [21] and *M*. *cuniculi* [22], isolated from cat, goat, horse and rabbit respectively. Moreover, four uncharacterized phylotypes were previously reported in healthy and psoriatic subjects [9,10] and, recently putative new *Malassezia* species were isolated from parrots and opossum [1], suggesting that *Malassezia* genus includes more species than currently known.

Despite the recent increase of studies focusing on *Malassezia* sp., little is still known concerning *Malassezia* genetic diversity. It has been proposed that specific *Malassezia* genotypes might be associated with skin diseases, such as atopic dermatitis [23] and also SD [24,25]. Moreover, limited information is available regarding *Malassezia* microbiota in Brazilian subjects based on molecular data. Therefore, this study aimed to characterize and compare *Malassezia* cutaneous microbiota from different body sites in Brazilian healthy subjects and patients with seborrheic dermatitis, based on *Malassezia* genetic diversity analysis.

## Materials and Methods

### Subjects and sample collection

This study was reviewed and approved by the Institutional Review Board from Pontifícia Universidade Católica de Campinas (PUC-Campinas), SP, Brazil (Protocol 604/08) and was conducted according to the principles expressed in the World Medical Association Declaration of Helsinki. Subjects were recruited in the Dermatology Department from Celso Pierro Hospital and Maternity, PUC-Campinas (Brazil). All subjects provided written informed consent prior to any study-related procedures. Exclusion criteria was comprised of antibiotic, antifungal or anti-dandruff therapy in the last 60 days prior to sampling; smoking; and the presence of cutaneous diseases that have been associated with *Malassezia* sp. except SD. To quantify SD severity, scalp desquamation, erythema and itching were all rated on a scale ranging from 0 to 5 points [26]. The values were totaled to indicate degrees of severity as follows: 0 points: healthy (no skin diseases related to *Malassezia* sp.); 1–5 points: mild SD; 6–9 points: moderate SD; 10–15 points: severe SD. Healthy (n = 5), mild SD (n = 5) and severe SD (n = 4) subjects were enrolled (S1 Table).

Samples from scalp and forehead were collected from each subject. No patient presented SD lesion on forehead. Non-scalp lesions from severe SD patients were also sampled (P02: chin, P10: shoulder; P14 and P22: interface between nape and hairline). In total, 32 samples were analyzed. Samples were collected using sterilized cotton swabs soaked in ST solution (0.15 M NaCl, 0.1% Tween 20) as previously described [10]. Two swabs were rubbed on the skin using zigzag repetitive movements, covering approximately 16 cm^2^ [12]. The heads of the swabs were cut, placed into microtubes containing ST solution, and centrifuged for 5 min [10]. Cotton swabs with no skin contact submitted to the same procedures were used as negative controls.

### DNA isolation and 5.8S/ ITS2 rDNA PCR amplification

Samples were vortexed with glass beads (Sigma-Aldrich, Saint Louis, MO, USA) in order to disrupt fungal cell walls. Total genomic DNA was extracted using DNeasy Blood & Tissue kit (Qiagen, Valencia, CA, USA) following the manufacturer’s recommendations. DNA was eluted in 50 μL and stored at -20°C. Negative controls were prepared using only ST solution subjected to the same procedures.

5.8S/ITS2 rDNA region was amplified using Mal1F (5′-TCTTTGAACGCACCTTGC-3′) and Mal1R (5′-AHAGCAAATGACGTATCATG-3′; H: A, T or C) *Malassezia*-specific primers [10]. Amplicon length ranges from 240 to 310 bp. PCR conditions were the same as described [10].

PCR products were excised from the agarose gel and purified using the QIAquick Gel Extraction Kit (Qiagen) following the manufacturer’s recommendations. Purified PCR products were eluted in 30 μl sterile water.

### Construction of PCR fragment libraries

Purified PCR products were cloned into pCR2.1 vector using TA Cloning Kit (Life Technologies, Carlsbad, CA, USA) following the manufacturer’s recommendations. *E*. *coli* DH5α competent cells were transformed using the total volume of ligation reaction and inoculated in LB-agar media containing ampicillin (100 mg/mL). Plates were incubated for 18 h at 37°C. Colonies from each library were picked using sterilized toothpicks, and added directly to PCR reaction tubes containing 1 X Taq Buffer, 1.25 U Taq polymerase (Thermo Scientific, Waltham, MA, USA), T7 and M13 vector primers (0.1 mM of each), 1.5 mM MgCl_2_, 0.25 mM of each dNTP and sterile water to a total volume of 50 μl. The following amplification conditions were used: initial denaturation (94°C, 2 min); 35 cycles comprising denaturation (94°C, 45 sec), annealing (50°C, 30 sec) and extension (72°C, 1 min); final extension (72°C, 2 min).

### Restriction fragment length polymorphism analyses (RFLP) and sequencing

**Fig 1:**
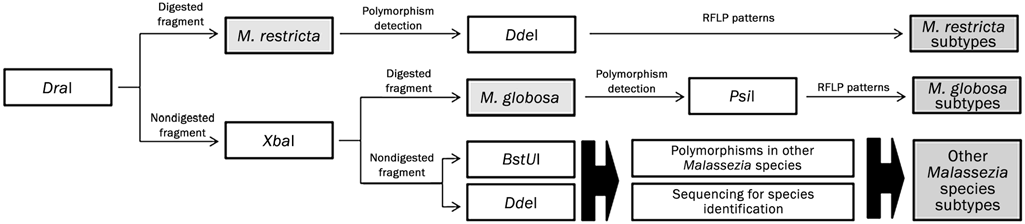
Restriction digestion steps. Digestion step sequence for *Malassezia* species identification and detection of intra-specific polymorphisms.

*In silico* restriction analysis was performed to select endonucleases capable of identifying *Malassezia* species and detecting intra-specific polymorphisms (Fig. 1). Restriction enzyme digestions were performed with 5 or 2.5 μL of PCR products and were done separately at 37°C for 60 or 90 min. Endonucleases were manufactured either by Promega (Madison, WI, EUA) or Thermo Scientific. Results were analyzed by electrophoresis on 2% agarose gels.

Representative clones from each *Malassezia* subtype were selected for sequencing. PCR products were purified using QIAquick PCR Purification Kit (Qiagen). Sequencing was performed using T7 primer utilizing ABI 3730 DNA Analyzer (Applied Biosystems, Foster City, CA, USA) at the Center for Study on Human Genome—University of São Paulo (Brazil).

### Sequence and Phylogenetic analysis

Identical sequences were grouped using BlastClust (http://toolkit.tuebingen.mpg.de/blastclust), and compared with GenBank database using BLAST algorithm [27].

Sequences were aligned using ClustalX 2.1 software (http://www.clustal.org/). Phylogenetic trees were constructed using Maximum Likelihood method and substitution model of Tamura 3-parameter utilizing Mega 5 software (http://www.megasoftware.net/). Ten-thousand bootstrap replications were performed. 5.8S/ITS2 rDNA sequences from *Malassezia* type strains (12 *Malassezia* species available in GenBank) were included.

### Statistical analysis

Clustering and non-metrical Multidimensional Scaling (nmMSD) using Bray-Curtis similarity distances [28] were performed to assess the relationships between *Malassezia* communities from different samples. Clustering analysis was done using SIMPROF test with 5% significance level. Individual species/subtypes contribution to microbiota groups was evaluated using SIMPER [29]. Analysis of Similarities (ANOSIM) test was applied to check differences based on health condition, body area and subjects [30]. ANOSIM global R value ranges from 1 to −1. R ≈ 1 indicates that intra-group variation is higher than inter-group variation. When R ≈ 0, there is the same level of variation within and between groups. Diversity was measured using Shannon-Weaver diversity index. These analyses were performed using Primer6 [29].

Species richness of each sample were evaluated using Chao 1 richness estimator [31]. Rarefaction plots were done to assess sampling sufficiency and community diversity analysis, using EstimateS [32].

For mean comparisons and variation analysis Two-tailed Student’s T-test and Pearson’s Variation Coefficient (CV) were performed, respectively.

## Results

### *Malassezia* species and subtypes in samples from healthy and SD subjects

Fifty to sixty clones from each skin sample were analyzed by RFLP, totaling 1763 clones. Mal1F and Mal1R primers anneal to conserved sites in 5.8S rDNA/ITS2 region, allowing the amplification of *Malassezia* organisms specifically. As this region is polymorphic within *Malassezia* genus, it is suitable for detecting intra-specific diversity, and also allows accurate species identification. Species assignment based on RFLP was confirmed by sequencing, and both methods yielded coherent results. In total, 778 sequences were obtained. Seventy-four sequences were chimeras, vector fragments, human DNA or poor quality sequences, and therefore were removed. Sequence analyses allowed species assignment and identification of subtypes that were not possible by RFLP.

**Fig 2:**
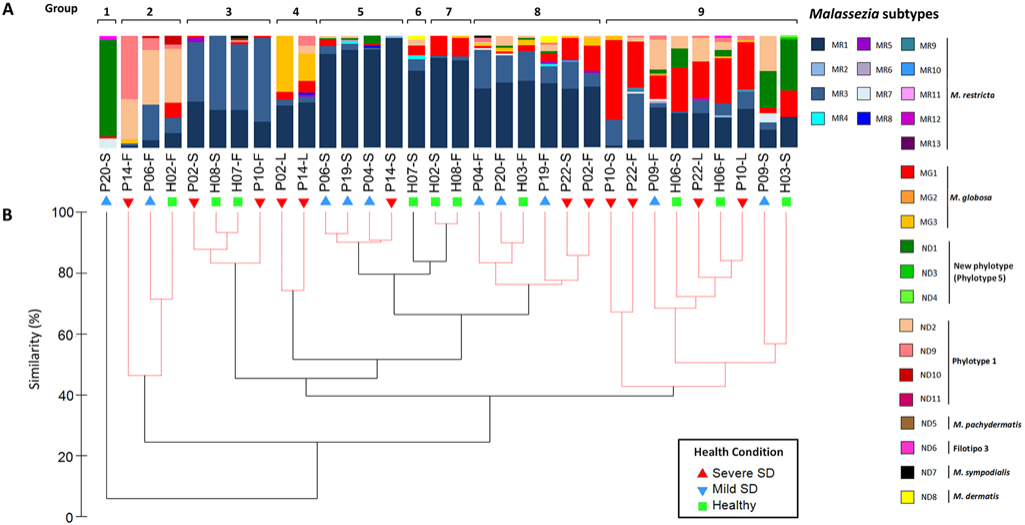
*Malassezia* subtype communities in 32 skin samples from healthy and SD subjects. (A) Proportion of *Malassezia* subtypes. (B) Clustering analysis of *Malassezia* microbiota. Solid black lines represent clusters significant at 95% according to Simprof test. Sample code: P: SD patient; H: healthy; S: scalp; F: forehead; L: non-scalp lesion sites.

Five known *Malassezia* species were detected. *M*. *restricta* was found in all samples, being the most abundant species in 22 samples (total 32 samples). *M*. *globosa* was detected in almost all samples (28/32), although it was the most abundant species in only 4 of them. *M*. *sympodialis*, (found in 2 samples) *M*. *dermatis* (2 samples) and *M*. *pachydermatis* (1 sample) were detected in low proportions. In total, 27 *Malassezia* subtypes were detected, mostly from *M*. *restricta* (13 subtypes), indicating variation among species and also intra-specific polymorphisms (Fig. 2A). Some subtypes are more common than others and others are rare on the analyzed samples. Subtypes MR1, MR3, MG1, ND2 and ND1 were the most abundant in samples. MR1 proportion is higher in scalp samples than in forehead ones and the opposite is found for MR3 proportions. *Malassezia* subtypes abundance varied depending on the sample. Subtypes of a same species occur in different proportions depending on the sample, suggesting that each sample has its own subtypes community.

**Fig 3:**
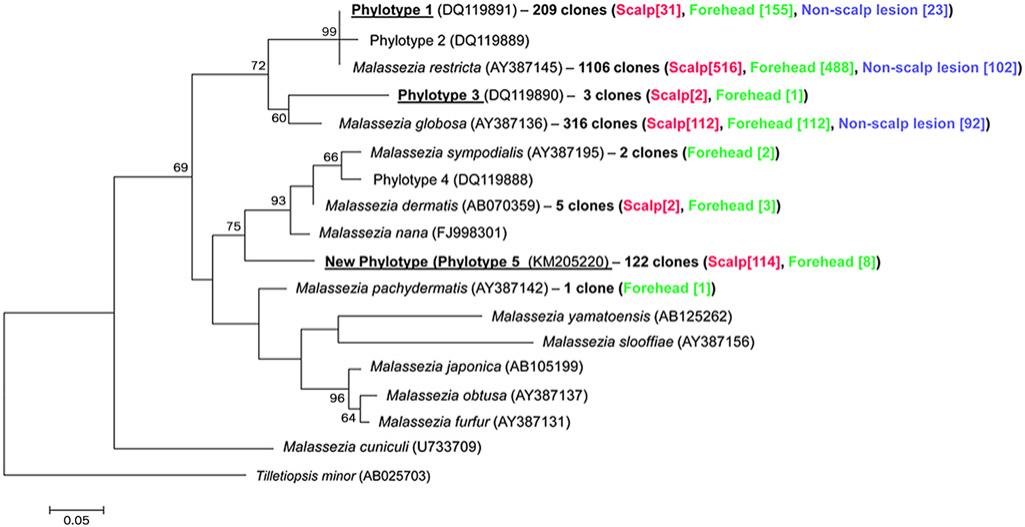
*Malassezia* phylogenetic tree. Maximum Likelihood phylogenetic tree based on 5.8S/ITS2 rDNA sequences from *Malassezia* sp. 5.8S/ITS2 rDNA sequences are available for 12 *Malassezia* species. Potential new *Malassezia* species found in this study are highlighted. Bootstrap values > 50% are shown. Codes between parentheses correspond to Genbank accession numbers. Numbers between brackets represent the number of *Malassezia* species clones by body site.

Three groups of sequences from uncharacterized organisms that might belong to potentially novel *Malassezia* species were found. Phylotypes 1 and 3 were previously detected in healthy subjects and patients with psoriasis [9,10] but were not characterized; and a new phylotype was first found on human skin in this work (Phylotype 5—nucleotide sequence deposited in the GenBank database with accession number KM205220), which did not cluster together with any formally described *Malassezia* species (Fig. 3). Phylotypes 1, 3 and 5 were detected in 20, 2 and 12 samples, respectively. Phylotype 1 was more abundant in forehead samples, whereas phylotype 5 predominated in scalp samples, and it was not detected in any samples from severe SD patients. Phylotype 1 was the most abundant organism in 3 samples, while in 3 other samples phylotype 5 was found in the highest proportion (Fig. 2A). Moreover, 5.8S/ITS2 rDNA fragment from phylotype 5 lengths 238 bp, being the smallest one among *Malassezia* genus sequences available for this region in GenBank which ranges from 240 to 310 bp. Different restriction subtypes were also detected for Phylotype 1 and Phylotype 5 (Fig. 2A).

### *Malassezia* community analysis

*Malassezia* communities from 32 skin samples were analyzed by Clustering, nmMDS and ANOSIM considering health status, body site and subject. Clustering analysis with SIMPROF test divided the *Malassezia* subtypes communities in 9 groups (Fig. 2B). Contribution of subtypes for group formation is shown in the (S2 Table).

*Malassezia* microbiota groups considering RLFP subtypes differ from the ones formed based on species abundance. At species level, only 6 groups are indicated by SIMPROF test (S1 Fig.). The reduction in the number of groups observed is not only due to group gathering when subtypes are counted together at species level, but also due to changes in the clustering dendogram topology. Species contribution to group formation is shown in S3 Table.

**Fig 4:**
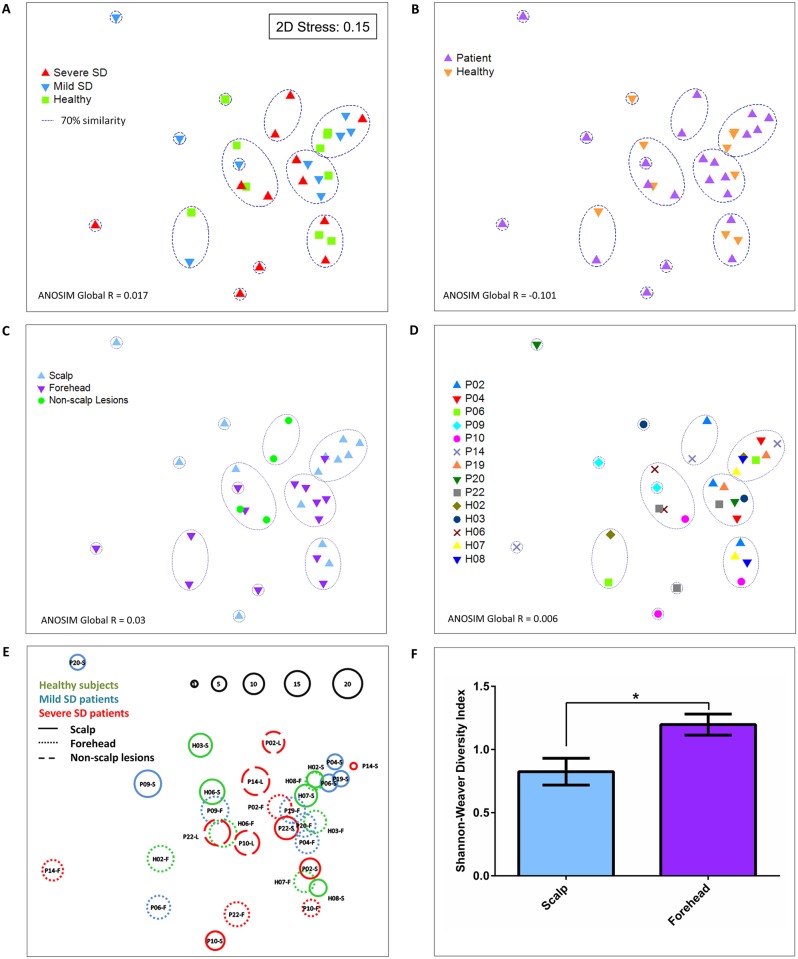
Multi Dimensional Scaling of *Malassezia* microbiota at subtype level in 32 skin samples from healthy subjects and SD patients. (A) Similarity between samples highlighting health condition specifying severity; (B) health condition not specifying severity; (C) body site and (D) subjects. (E) Sample diversity. Circle diameter is proportional to the Shannon-Weaver index. Bubble size scale indicates the relative diversity, with the smallest diversity (P14-S) indexed as 1. (F) Mean diversity based on Shannon-Weaver diversity indexed by body site. Student’s T-test with bars representing Mean ± SEM. * p < 0.05. Sample code: P: SD patient; H: healthy; S: scalp; F: forehead; L: non-scalp lesion sites.

Considering health condition specifying disease severity (severe or mild SD), no grouping of samples was observed by clustering or nmMSD (Fig. 4A). This is also supported by ANOSIM test (R = 0.017). Similar results were obtained when SD severity was not specified (ANOSIM R = -0.101; Fig. 4B) Moreover, no dichotomy between *Malassezia* microbiota from scalp and forehead samples was detected (R = 0.03; Fig. 4C). Samples from different body areas from the same subject did not group preferentially either (global R = 0.006; Fig. 4D), indicating that each sample has its own specific microbiota.

*Malassezia* diversity varied among samples (Fig. 4E and S2 Fig.). For the majority of samples, rarefaction curves reached plateaus, indicating that sample diversity was sufficiently explored (S2 Fig.). Species richness estimated by Chao1 is greater than the observed richness, suggesting the presence of rare subtypes that were not covered in sampling effort (S2 Fig.). Most scalp samples are less diverse in comparison to forehead samples. Diversity measured by Shannon-Weaver index considering samples by body site shows similar results (Two-Tailed Student’s T-test p = 0.0103), even though in scalp samples are included samples from healthy subjects and from SD patients (Fig. 4F).

Analyzing each organism individually, relative data variation for *M*. *globosa* proportions was compared considering Pearson’s coefficient of variation (CV). Scalp and forehead coefficients are 5.4 (CV = 1.41) and 6.2 (CV = 1.64) times higher, respectively, than the non-scalp lesion sites coefficient (CV = 0.26), indicating that samples from these areas present more variation than the non-scalp lesions. *M*. *globosa* mean proportion in the non-scalp lesion sites is 42.28%, which is larger than proportions found in most samples from scalp and forehead. This suggests a closer association of *M*. *globosa* with lesions from areas other than scalp from severe SD patients. *M*. *globosa* also predominated in two other samples from severe SD patients (scalp from subject P10 and forehead from subject P22 (Fig. 2A).

## Discussion

*Malassezia* genus have been widely studied in recent years due to their association with skin diseases. Different aspects have been analyzed, such as the specific fungal roles on the pathogenic process [24,25,33–35] and the host immune response [36–42]. The genomic sequences of *M*. *globosa* [33] and *M*. *sympodialis* [43] have been completely determined, and the partial sequence of *M*. *restricta* is also available [33]. Despite advances, the role that *Malassezia* yeasts play in the pathogenic processes is still poorly understood.

Studies based on molecular methods in different populations frequently show *M*. *restricta* as the most abundant *Malassezia* species on healthy and diseased human skin, followed by *M*. *globosa* [9–13,44–46]. In our study with Brazilian subjects, *M*. *restricta* was detected in all samples, and was the most abundant species in the majority of them. *M*. *globosa* was detected in most samples, although in low proportions. Other formally described species such as *M*. *sympodialis*, *M*. *pachydermatis* and *M*. *dermatis* were found in few samples and in low proportions in this study, and *M*. *furfur* was not detected. *M*. *furfur* has been shown to be rare or even absent, and when detected, is found in low proportions by molecular-based studies [3,12,47–50]. In contrast, it is often isolated by culture-based methods [51–53], which favor faster-growing organisms like *M*. *furfur*. In culture, this species normally overtakes fastidious species such as *M*. *restricta* [54,55]. In this study, rarefaction curves were performed to verify the sampling effort of the clone libraries, and the results indicated that the observed diversity approximates or equals the estimated one. Additionally, PCR amplification tests using Mal1F/Mal1R primer set successfully amplified *Malassezia* type strains and clinical isolates, including *M*. *furfur*, thus indicating that the adopted methodology would allow the detection of this species.

Three groups of sequences that might belong to uncharacterized organisms were detected, two of them previously found in healthy subjects and patients with psoriasis [9,10]. Here, we report that phylotype 1 was detected in most samples, found in higher proportions in forehead samples and being the most prevalent organism in some of them. In contrast, Paulino *et*. *al*, 2006 reported that phylotype 1, although present in many studied samples, was found in low proportions. Additionally, a new phylotype (phylotype 5) was found in the Brazilian subjects. This new phylotype did not cluster with any other formally described species and has sequence identity <85% comparing to other *Malassezia* sp. sequences, suggesting that it might indeed be a new *Malassezia* species. Further studies are necessary in order to isolate, characterize and formally describe these putative new taxa, including molecular analysis of other genomic regions such as ribosomal large subunit D1/D2, as well as morphological and biochemical analyses. Phylotype 5 was found preferentially in scalp samples rather than in forehead, being the predominant organism in 3 samples. It is important to note that it was not detected in any sample from severe SD patients, suggesting that it inhabits preferentially skin from healthy and mild SD subjects. This might be related to immune response on severe SD subjects, which can potentially interfere with its growth and maintenance on skin.

Several *Malassezia* subtypes were identified, indicating high intra-specific diversity. Subtype proportions varied between samples, resulting in differences in clustering dendogram topologies at subtype level in comparison with species level. This suggests that *Malassezia* communities might vary between samples even when species proportions are similar. Therefore, studying *Malassezia* microbiota at intra-specific level may be useful to identify genotypes associated with skin diseases.

Samples did not group according to health condition, nor to body area and subject, and no *Malassezia* genotype was detected preferentially on SD patients. These findings are interesting, as they differ from other studies that reported specific *Malassezia* genotypes associated with SD [24,25]. DNA regions varied between studies, which might be related to the differences observed. We found that *Malassezia* diversity is higher in forehead than in scalp samples, regardless scalp health status, which suggests diversity patterns associated with body site and not with health condition. Although no dichotomy according to health status was observed, it is possible that specific lineages might be more closely involved with SD pathogenic process. Other possible markers might help to elucidate this association.

Considering the relative abundance of each *Malassezia* organism individually *M*. *globosa* occurred in high proportions in all samples from non-scalp lesions from severe SD patients. High *M*. *globosa* proportions were also found in some of the scalp and forehead samples from severe SD patients, suggesting that this species might be more closely related to SD. Previous studies suggested that *M*. *globosa* is associated with SD [24,33,45,56,57]; however, they do not differentiate disease occurrence in scalp and other body areas. We found different *M*. *globosa* proportions in non-scalp lesions as compared with scalp lesions from mild and severe SD patients, suggesting that *M*. *globosa* might be associated with SD symptoms from areas other than scalp. Perhaps this species is more efficient in evading and modulating local immune response so it can grow in these areas, despite inflammation occurrence.

*Malassezia* sp. has been associated with SD and it is known that only some individuals develop SD [6], suggesting that both yeasts and host plays a role in SD development. We observed no group formation according to health condition, indicating that other factors aside from *Malassezia* can be involved in skin diseases development, such as immune system response [58–60]. In healthy individuals, probably host immune system controls *Malassezia* growth, allowing its maintenance without local inflammation, reaching an equilibrium on human skin [6]. Factors that turn *Malassezia* into a pathogen are not yet fully understood [6]. Possibly the ability of these fungi to modulate local immune response allied to host susceptibility and second metabolites production have a role in SD development [2]. High lipase gene expression and lipolytic activity of *M*. *globosa* lipases may also be associated with SD development [35,56,61]. The correlation of severe SD cases with AIDS patients suggests that host immune response indeed has an important role on SD [2,58,62]. In this context, differential gene expression of immune response genes from healthy and SD patients has been reported [60]. Patients treated with antifungal drug exhibited gene expression pattern similar to healthy subjects, supporting the relation between host gene expression and skin disease development.

In Brazil, most data regarding *Malassezia* organisms are provided by veterinary studies [63–67]. To our knowledge, studies aiming to analyze *Malassezia* microbiota in Brazilian population are based on culture-dependent methods [68,69]. The same happens in other Latin American countries [52,70–72]. Currently, culture-independent methods are preferentially chosen due to culture bias and the fastidious nature of many microorganisms, including *Malassezia* [10,45,54,73–75]. Thus the available information regarding *Malassezia* microbiota in Brazil is still restricted and possibly influenced by limitations of culture-dependent methods.

Our data show a general view of *Malassezia* microbiota in healthy and SD subjects through culture-independent methods in Brazilian subjects, pointing to particularities of the analyzed population. The number of subjects included in the study is limited, and is not nearly sufficient to understand the complexity of the *Malassezia* microbiota in human skin. Nevertheless, the data reported here establishes the basis for future studies, which should include a higher number of subjects to provide a broader understanding of *Malassezia* microbiota in Brazilian population and the role of these organisms as commensals and as pathogens. Three groups of sequences from uncharacterized organisms that might belong to new *Malassezia* species were detected in samples from both healthy and SD subjects. One of the organisms had not been previously reported on human skin, and was mainly found in scalp samples. We intend to confirm the taxonomic status and formally describe this putative new taxon. These findings suggest differences between studies, and indicate that *Malassezia* diversity from human skin has not been fully explored. Differences observed in samples from Brazilian subjects might be associated with genetics and cultural habits, such as hair care. Hair appearance is an important cultural feature in Brazil and different products such as gels, creams or chemical treatments for dyeing and smoothing are commonly used. Such habits can impact the microbiota.

Our results do not suggest an association between *Malassezia* microbiota and SD, body area or subject based on the whole *Malassezia* community structure, but indicate less diversity in scalp than forehead. At organism level, higher *M*. *globosa* proportions in non-scalp lesions in severe SD subjects were found, suggesting closer association of this species with SD lesions from areas other than scalp. In order to confirm whether *M*. *globosa* plays a role in SD specifically in body areas other than scalp, it would be important to increase the number of samples analyzed in further studies. It would also be important to analyze *M*. *globosa* lipase gene expression and enzyme activity, as well as the host immune response. Different *Malassezia* subtypes were found in different proportions in samples. This shows that *Malassezia* communities can differ at intra-specific level, but can be similar at species level. Other factors beyond *Malassezia* presence, species, subtypes and diversity patterns might have an important role in SD development, such as genetics and host immune response.

Here we showed the first panorama of *Malassezia* microbiota from Brazilian subjects based on molecular methods. Results revealed potential new species occurring in large proportions in most samples. Different subtypes of *Malassezia* species were found, demonstrating that analyses aiming to explore intra-specific diversity in depth are a significant motivation for further studies to elucidate *Malassezia* role in skin diseases.

## Supporting Information

S1 Fig *Malassezia* communities from 32 skin samples from healthy and SD subjects at species level. (A) *Malassezia* species and phylotypes proportion in each sample. (B) Clustering analysis of *Malassezia* microbiota. Solid black lines represent clusters significant at 95% according to Simprof test. Sample code: P: SD patient; H: healthy; S: scalp; F: forehead; L: non-scalp lesions.
(TIF)

S2 Fig Rarefaction curves at *Malassezia* subtype level. Sample code: P: SD patient; H: healthy; S: scalp; F: forehead; L: Non-scalp lesion sites. Sobs: observed subtypes. Chao1: Estimated richness. Doted lines represent Standard Deviation.
(TIF)

S1 Table Subjects included in this study. (DOCX)

S2 Table Contribution of *Malassezia* subtypes. Contribution for groups formed by clustering analysis with SIMPROF test. Higher contributing subtypes (cut-off 90%) are listed.
(DOCX)

S3 Table Contribution of *Malassezia* species or phylotypes. Contribution for groups formed by clustering analysis with SIMPROF test. Higher contributing subtypes (cut-off 90%) are listed.
(DOCX)
