# Supplementary figures and images for: Malassezia Intra-Specific Diversity and Potentially New Species in the Skin Microbiota from Brazilian Healthy Subjects and Seborrheic Dermatitis Patients
Source: PLoS One. 2015 Feb 19;10(2):e0117921. doi: 10.1371/journal.pone.0117921 (PMC4335070; doi:10.1371/journal.pone.0117921)

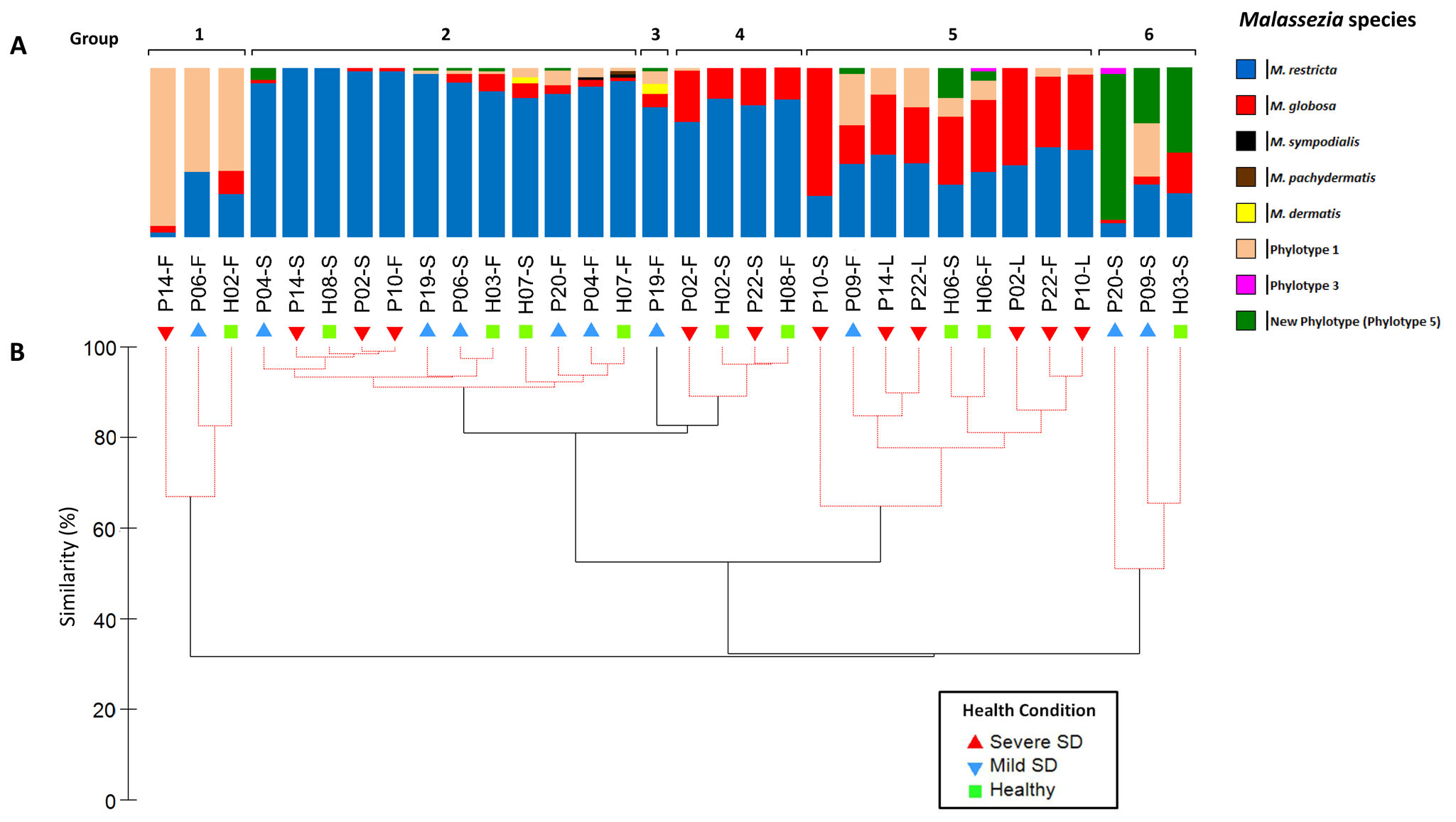

Supplement: S1 Fig — (A) Malassezia species and phylotypes proportion in each sample. (B) Clustering analysis of Malassezia microbiota. Solid black lines represent clusters significant at 95% according to Simprof test. Sample code: P: SD patient; H: healthy; S: scalp; F: forehead; L: non-scalp lesions. (TIF) [file pone.0117921.s001.tif]

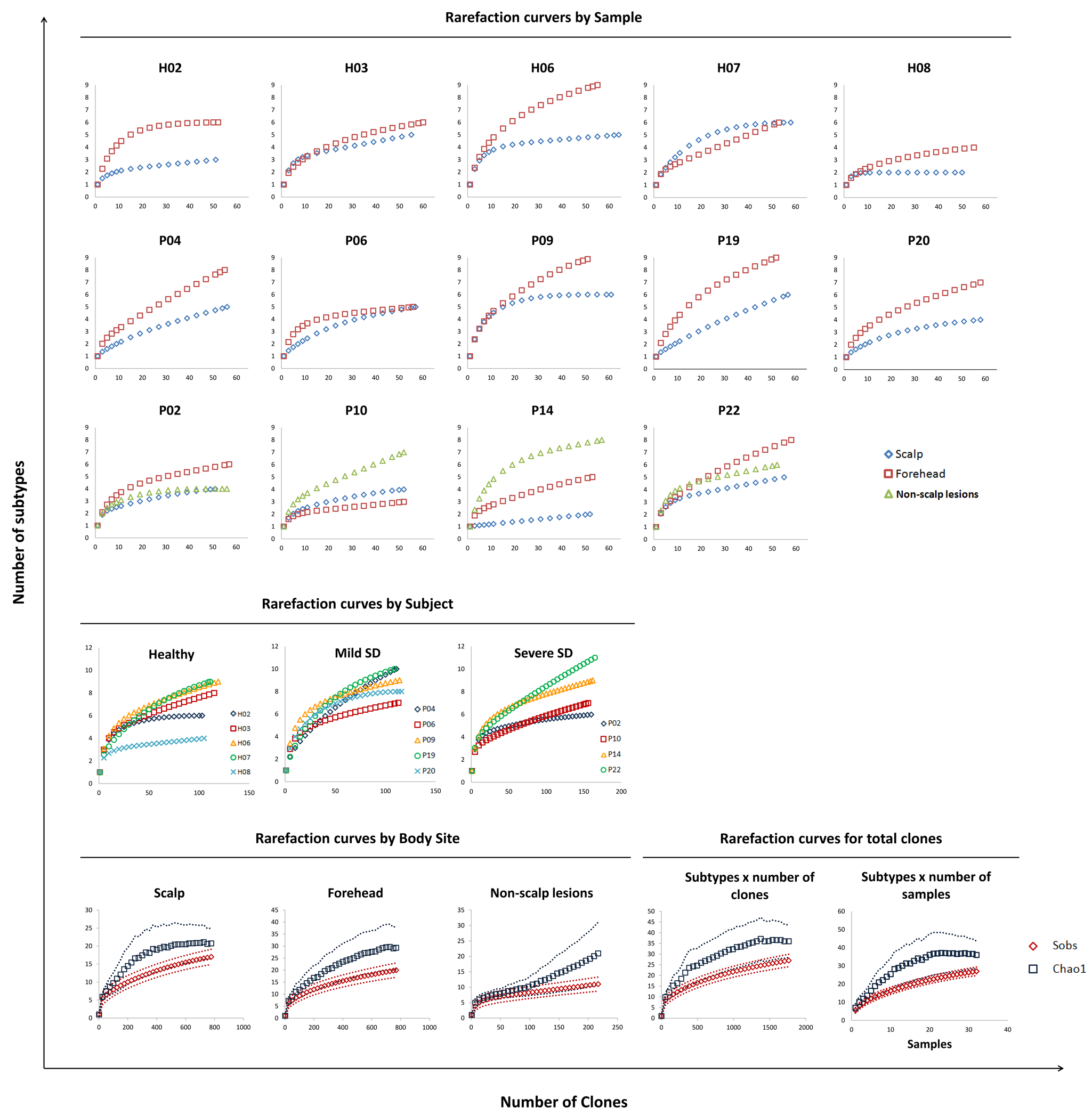

Supplement: S2 Fig — Sample code: P: SD patient; H: healthy; S: scalp; F: forehead; L: Non-scalp lesion sites. Sobs: observed subtypes. Chao1: Estimated richness. Doted lines represent Standard Deviation. (TIF) [file pone.0117921.s002.tif]
