# Supplementary material for: Malassezia Intra-Specific Diversity and Potentially New Species in the Skin Microbiota from Brazilian Healthy Subjects and Seborrheic Dermatitis Patients
Source: PLoS One. 2015 Feb 19;10(2):e0117921. doi: 10.1371/journal.pone.0117921 (PMC4335070; doi:10.1371/journal.pone.0117921)
Supplement: S1 Table — (DOCX) [file pone.0117921.s003.docx]

**Table S1:** **Subjects included in this study.**

| **Subject** | **Health Condition (Score)**^a^ | **Gender** | **Age group (years)** | **Ethnic group** |
| --- | --- | --- | --- | --- |
| H02 | Healthy (0) | F | 21-30 | Caucasian |
| H03 | Healthy (0) | F | 21-30 | African descent |
| H06 | Healthy (0) | F | 51-60 | Caucasian |
| H07 | Healthy (0) | M | 21-30 | Caucasian |
| H08 | Healthy (0) | M | 31-40 | Caucasian |
| P04 | Mild SD (5) | F | 31-40 | African descent |
| P06 | Mild SD (5) | F | 41-50 | African descent |
| P09 | Mild SD (5) | F | 31-40 | Caucasian |
| P19 | Mild SD (3) | F | 31-40 | Biracial |
| P20 | Mild SD (4) | F | 41-50 | Caucasian |
| P02 | Severe SD (10) | F | 41-50 | Caucasian |
| P10 | Severe SD (12) | M | 31-40 | Caucasian |
| P14 | Severe SD (11) | M | 21-30 | African descent |
| P22 | Severe SD (11) | F | 31-40 | Biracial |

M: male; F: female

^a^Score corresponds to the point scale adopted to classify subjects according to health condition
