# Supplementary material for: Malassezia Intra-Specific Diversity and Potentially New Species in the Skin Microbiota from Brazilian Healthy Subjects and Seborrheic Dermatitis Patients
Source: PLoS One. 2015 Feb 19;10(2):e0117921. doi: 10.1371/journal.pone.0117921 (PMC4335070; doi:10.1371/journal.pone.0117921)
Supplement: S2 Table — Contribution for groups formed by clustering analysis with SIMPROF test. Higher contributing subtypes (cut-off 90%) are listed. (DOCX) [file pone.0117921.s004.docx]

**Table S2:** **Contribution of *Malassezia* subtypes for groups formed by clustering analysis with SIMPROF test. Higher contributing subtypes (cut-off 90%) are listed.**

|  |  | **Primary contribution** | | **Secondary contribution** | | **Tertiary contribution** | |
| --- | --- | --- | --- | --- | --- | --- | --- |
| **Group**^a^ | **Mean Similarity (%)** | **Subtype** | **Contribution (%)** | **Subtype** | **Contribution (%)** | **Subtype** | **Contribution (%)** |
| 2 | 54.82 | ND2 | 70.9 | ND9 | 11.18 | MR3 | 10.07 |
| 3 | 86.42 | MR3 | 65.91 | MR1 | 32.97 | - | - |
| 4 | 74.34 | MR1 | 50.00 | MG3 | 33.33 | MG1 | 9.52 |
| 5 | 90.68 | MR1 | 96.76 |  |  |  |  |
| 7 | 96.23 | MR1 | 80.39 | MG1 | 17.65 | - | - |
| 8 | 79.00 | MR1 | 67.17 | MR3 | 22.47 | MG1 | 5.58 |
| 9 | 54.53 | MG1 | 45.17 | MR1 | 30.53 | MR3 | 10.13 |

^a^ Numbered according to Figure 2B. Groups 1 and 6 were not included since they comprise only one sample each.
