# Supplementary material for: Malassezia Intra-Specific Diversity and Potentially New Species in the Skin Microbiota from Brazilian Healthy Subjects and Seborrheic Dermatitis Patients
Source: PLoS One. 2015 Feb 19;10(2):e0117921. doi: 10.1371/journal.pone.0117921 (PMC4335070; doi:10.1371/journal.pone.0117921)
Supplement: S3 Table — Contribution for groups formed by clustering analysis with SIMPROF test. Higher contributing subtypes (cut-off 90%) are listed. (DOCX) [file pone.0117921.s005.docx]

**Table S3:** **Contribution of *Malassezia* species or phylotypes for groups formed by clustering analysis with SIMPROF test. Higher contributing subtypes (cut-off 90%) are listed.**

|  |  | **Primary contribution** | | **Secondary contribution** | |
| --- | --- | --- | --- | --- | --- |
| **Group**^a^ | **Mean Similarity (%)** | **Species** | **Contribution (%)** | **Species** | **Contribution (%)** |
| 1 | 72.17 | Phylotype 1 | 82.94 | *M. restrica* | 15.3 |
| 2 | 92.87 | *M. restricta* | 97.35 | - | - |
| 4 | 92.71 | *M. restricta* | 80.57 | *M. globosa* | 19.43 |
| 5 | 77.37 | *M. restricta* | 48.29 | *M. globosa* | 45.1 |
| 6 | 55.87 | Phylotype 5 | 69.13 | *M. restrica* | 25.72 |

^a^ Numbered according to Figure S2. Group 3 was not included since they comprise only one sample each.
